# Microstructure of Ni_0.5_Zn_0.5_Fe_2_O_4_ Nanofiber with Metal Nitrates in Electrospinning Precursor

**DOI:** 10.3390/nano10071344

**Published:** 2020-07-09

**Authors:** Kyeong-Han Na, Wan-Tae Kim, Tae-Hyeob Song, Sung-Wook Kim, Won-Youl Choi

**Affiliations:** 1 Department of Advanced Materials Engineering, Gangneung-Wonju National University, Gangneung 25457, Korea; nag0717@naver.com (K.-H.N.); dktkzz1@naver.com (W.-T.K.); 2 Korea Institute of Civil Engineering and Building Technology, Goyang 10223, Korea; thsong@kict.re.kr (T.-H.S.); swkim@kict.re.kr (S.-W.K.); 3 Research Institute for Dental Engineering, Gangneung-Wonju National University, Gangneung 25457, Korea

**Keywords:** nanofibers, electrospinning, diameter control, NiZn ferrite

## Abstract

Electrospun NiZn ferrite nanofibers have great potential due to their one-dimensional structure and electrical properties, but they have a low reproducibility resulting from many process confounders, so much research effort is needed to achieve optimized process control. For structure control, the viscosity of the precursor solution is a likely parameter. One solution is to use polyvinyl pyrrolidone (PVP) and metal nitrate to obtain the desired viscosity by increasing the nitrate content, even if the polymer content is decreased. Ni_0.5_Zn_0.5_Fe_2_O_4_ ferrite nanofiber was electrospun with various precursor conditions. Fifteen different precursor solutions, with a content of five polymers and three metal nitrates, were prepared, with precursor solutions composed of Fe(NO_3_)_2_·9H_2_O, Ni(NO_3_)_2_·6H_2_O, Zn(NO_3_)_2_·6H_2_O, polyvinyl pyrrolidone (PVP), and N,N-dimethylmethanamide. The fiber diameter changed from the lowest, of 62.41 nm, to 417.54 nm. This study shows that the average diameter can be controlled using the metal nitrate concentration without a difference in crystal structure when PVP is used. In a 24.0 mmol metal nitrate precursor solution, the process yield was improved to 140% after heat treatment. There was also no significant difference in the crystal structure and morphology. This system reduces the cost of raw materials for electrospinning and increases the process yield of NiZn ferrite nanofibers.

## 1. Introduction

Ever since nanoscale materials have been synthesized and applied, the inactive effects have been ignored for the bulk state, but can no longer be disregarded. For example, as the material size decreases, the surface area increases rapidly, and the bandgap transition also increases. The optical transition, the increase in catalytic activity, and the improvement in electrical conductivity were the results of previous studies [1,2,3,4]. Due to thermal fluctuations, ferromagnetic materials become superparamagnetic [5]. To make these effects useful, many studies have been carried out in various fields, and diverse nanostructures have been suggested according to application directions [6,7,8,9,10]. Among them, one-dimensional (1-D) nanostructures, such as nanofibers, nanotubes, and nanorods, have been proposed for applications that need superior electrical properties, like photo-electronic devices, sensors, batteries, and actuators, because of their large surface area and improved electron mobility [11,12,13]. Because the A_x_B_1−x_Fe_2_O_4_ spinel ferrite phases, including substitutional atoms like Ni, Zn, Mn, and Co, are generally studied for electrode materials with magnetic properties, many experiments have tried to apply a 1-D nanostructure for the spinel ferrite [14,15,16,17]. However, since the size reduction of the nanostructure increases the magnetically dead layer ratio [18,19], a technique is required to control the diameter in order to optimize the size. There are various synthesis methods for one-dimensional nanostructures, such as self-assembly, anodizing, hydrothermal, and electrospinning [20,21,22,23,24,25]. Among them, the electrospinning method can be applied to the nanofibers of various chemical compositions, requires simple equipment components, and has a low initial cost. However, since the electrospinning process is too sensitive to environmental changes, including temperature and humidity, and has poor reproducibility, it is essential to obtain meticulous process control, based on enough experimental data. To control the average diameter of the electrospun nanofibers, an effective method to change the viscosity of the precursor solution is needed. With a solution that uses polyvinyl pyrrolidone (PVP) and metal nitrates at the same time, the viscosity increases rapidly, according to the content of the nitrates, and can be used to control the diameter of nanofibers while reducing the PVP usage [26].

Since previously the average diameter of nanofibers was controlled by changing the polymer content, the following experiment was carried out to compare the difference in microstructure between the diameter being controlled by the polymer content versus the nitrate content [26]. First, PVP and N,N-dimethylmethanamide (DMF) solutions, with five different compositions, were prepared, and then three different weights of Ni, Zn, and Fe nitrate were added to each polymer solution. The 15 different precursor solutions were prepared and electrospun for the comparison of microstructures. Before electrospinning, a tensiometer and a viscometer were used to measure the surface tension and viscosity of the precursor solutions, respectively. The heat treatment was set up according to the temperature calculated through thermogravimetric–differential thermal analysis (TGA-DTA) for the as-spun nanofibers, and the average diameter of the nanofibers fabricated with each set of conditions was measured using field-emission scanning electron microscope (FE-SEM) image analysis. We used X-ray diffraction (XRD) analysis to determine whether or not the crystal phase changed according to the content of metal nitrates.

## 2. Materials and Methods

The reagents used to prepare the precursor solutions were as follows: Ni(NO_3_)_2_·6H_2_O (EP, Samchun Chemicals Co. Ltd., Seoul, Korea), Zn(NO_3_)_2_·6H_2_O (EP, Daejung Chemicals Co. Ltd., Gyeonggi, Korea), Fe(NO_3_)_2_·9H_2_O (GR, Kanto Chemical Co. Inc., Tokyo, Japan), Polyvinyl pyrrolidone (PVP, M.W. 1,300,000, Alfa Aesar Korea Co. Ltd., Incheon, Korea), and N,N-dimethylmethanamide (DMF, EP, Daejung Chemicals Co. Ltd., Gyeonggi, Korea). In this experiment, all of the materials were calculated by weight ratio. To prepare the polymer solutions, PVP was added to DMF at concentrations of 10 wt%, 12.5 wt%, 15 wt%, 17.5 wt%, and 20 wt%, and then stirred using a magnetic stirrer for 48 h. After that, mixed nitrate powder in quantities of 4.8 mmol, 14.4 mmol, and 24 mmol, with a molar ratio of Ni(NO_3_)_2_·6H_2_O:Zn(NO_3_)_2_·6H_2_O:Fe(NO_3_)_2_·9H_2_O = 1:1:4, was added to 100 g of each polymer solution and stirred until completely dissolved. Each precursor solution was labeled as polymer concentration—A, B, and C. A, B, and C refer to metal nitrate inputs of 4.8 mmol, 14.4 mmol, and 24 mmol, respectively. For example, precursor solution 15-B denotes a sample in which 14.4 mmol of metal nitrate is added to 100 g of 15 wt% PVP/DMF solution. The composition of each precursor solution is shown in Table 1.

**Figure 1:**
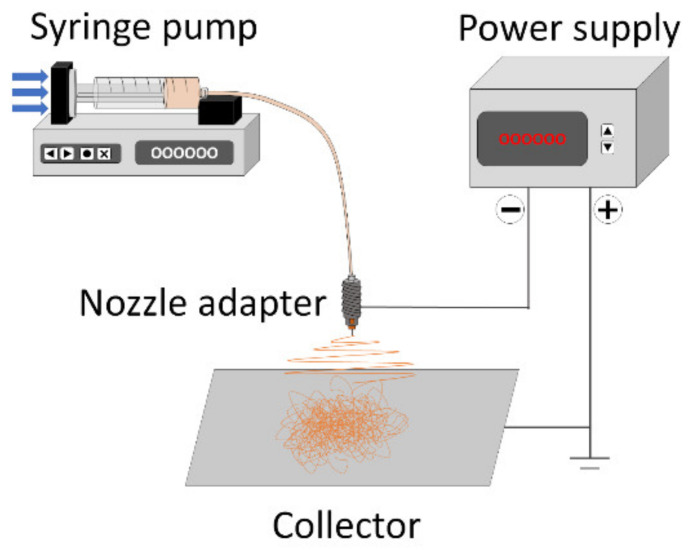
Scheme of the electrospinning equipment components.

The 15 different precursor solutions were used to fabricate nanofibers through the following typical electrospinning process. The precursor solutions were loaded into a 12 mL polypropylene syringe with a 15.56 mm diameter and mounted on a syringe pump. The syringe was connected to a stainless steel nozzle adapter held on a holder using luer lock tubing, and a 16-gauge capillary was attached to the end of the nozzle adapter. The nozzle adapter was connected to a power supply to apply a high voltage, and a flat aluminum foil collector was located 20 cm below the tip of the capillary. The collector was grounded using copper cable. The scheme of the equipment setup is as shown in Figure 1.

A voltage of 20 kV was applied to the nozzle adapter while a solution, at a flow rate of 0.2 mL/h, was continuously emitted using a syringe pump. To control the confounder, the environmental conditions were maintained at room temperature and at a relative humidity of less than 40%. The nanofibers collected from the plate were separated from the foil and dried at 80 °C for 2 h using a dry oven. Next, the dried as-spun nanofiber sample was heat treated for 3 h at a temperature of 650 °C, and a heating rate of 5 °C/min, in atmospheric gas to evaporate all volatile components and effect phase transformation to crystallized NiZn ferrite nanofibers.

The viscosity and surface tension of the precursor solutions were obtained using a tensiometer (K100, KRUSS, Hamburg, Germany) and viscometer (LVT, AMETEK Brookfield, Middleboro, MA, USA), and the Wilhelmy plate method was used to measure the surface tension. The morphology and average diameter of the as-spun and calcined nanofibers were measured by field-emission scanning electron microscope (FE-SEM) (SU-70, Hitachi Co. Ltd., Niigata, Japan) image analysis. Thermogravimetric—differential thermal analysis (TGA-DTA) was carried out to obtain the optimized heat treatment conditions for the as-spun nanofibers. The TGA-DTA analysis was measured from 20 °C to 800 °C in atmospheric gas and at a heating rate of 5 °C/min using the equipment (STA 409, NETZSCH, Selb, Germany). To confirm the crystal structure of the nanofibers, X-ray diffraction (XRD) was carried out using an X-ray diffractometer (Empyrean, Malvern Panalytical, Almelo, Nederland), and K-alpha 1 and K-alpha 2 of the Cu target were used as light sources. A generator voltage of 40 kV and a tube current of 40 mA were applied to the Cu target. The K-alpha 2/K-alpha 1 ratio was set to 0.5, and the diffraction pattern was obtained from 20° to 70° at 0.0065° per step.

The morphology and average diameter of the nanofibers were confirmed by FE-SEM image analysis. The high-temperature behavior of the as-spun nanofibers was confirmed by TGA-DTA analysis, and the crystal structure of calcined nanofibers was confirmed by comparing XRD results with joint committee on powder diffraction standards (JCPDS) cards.

## 3. Results and Discussion

### 3.1. Surface Tension and Viscosity of the Precursor Solutions

**Figure 2:**
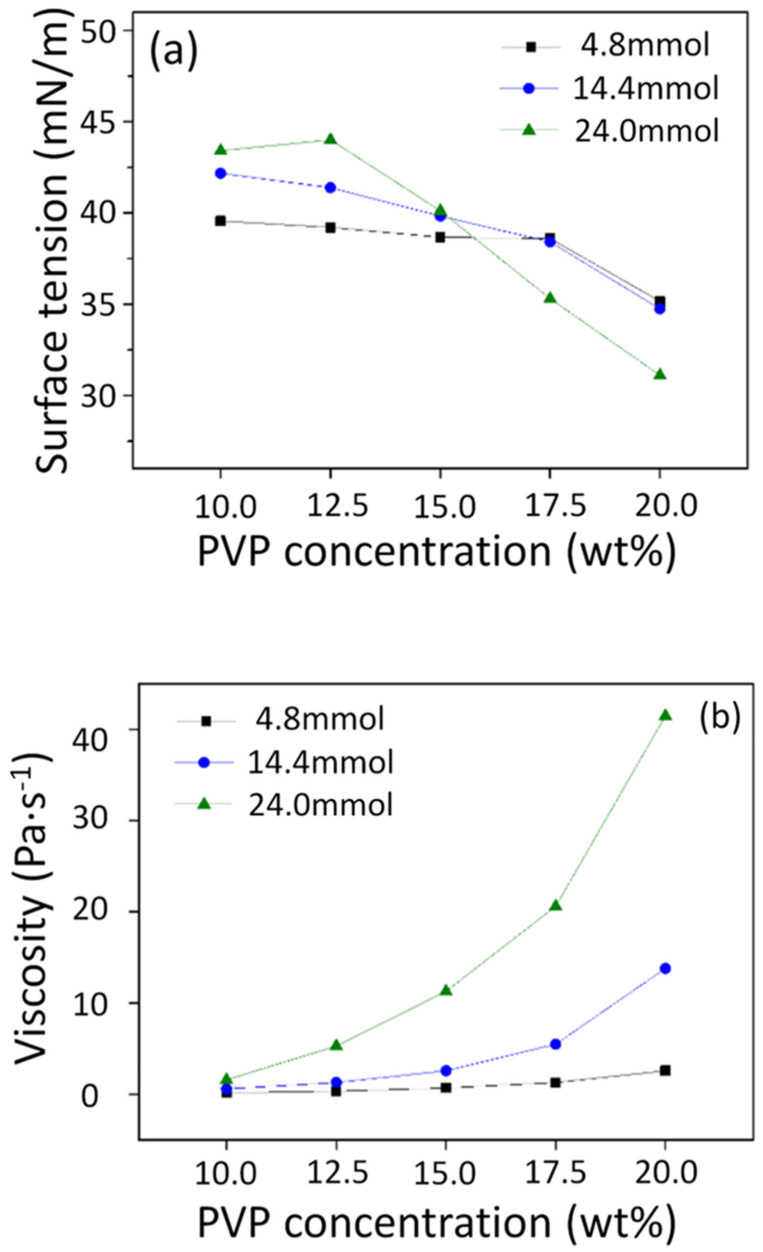
Surface tension (**a**) and viscosity (**b**) of each precursor solution.

The surface tension and viscosity of the prepared precursor solutions are shown in Figure 2. According to the surface tension change shown in Figure 2a, the surface tension in 4.8 mmol, 14.4 mmol, and 24.0 mmol of metal nitrates decreased with the PVP concentration. The surface tension was proportionally increased with the metal nitrates in the low PVP concentration, but at a high PVP concentration, it was inversely proportionally decreased. The suppression of the surface tension may be a factor that decreases the critical voltage for the jet formation in electrospinning. Figure 2b shows the viscosity tendency of each precursor solution, which confirms that the viscosity of the solution rapidly increased with both PVP and metal nitrates content. Considering that the average diameter of the nanofibers obtained via electrospinning is proportional to the viscosity of the precursor solution, the desired diameter can be obtained by reducing the polymeric support content and increasing the content of metal nitrates. PVP is a component that volatilizes; however, inorganic components of metal nitrates remained after the heat treatment process, so a precursor solution with a low PVP concentration can increase the yield of the process and reduce the production cost.

### 3.2. As-Spun Nanofibers

**Figure 3:**
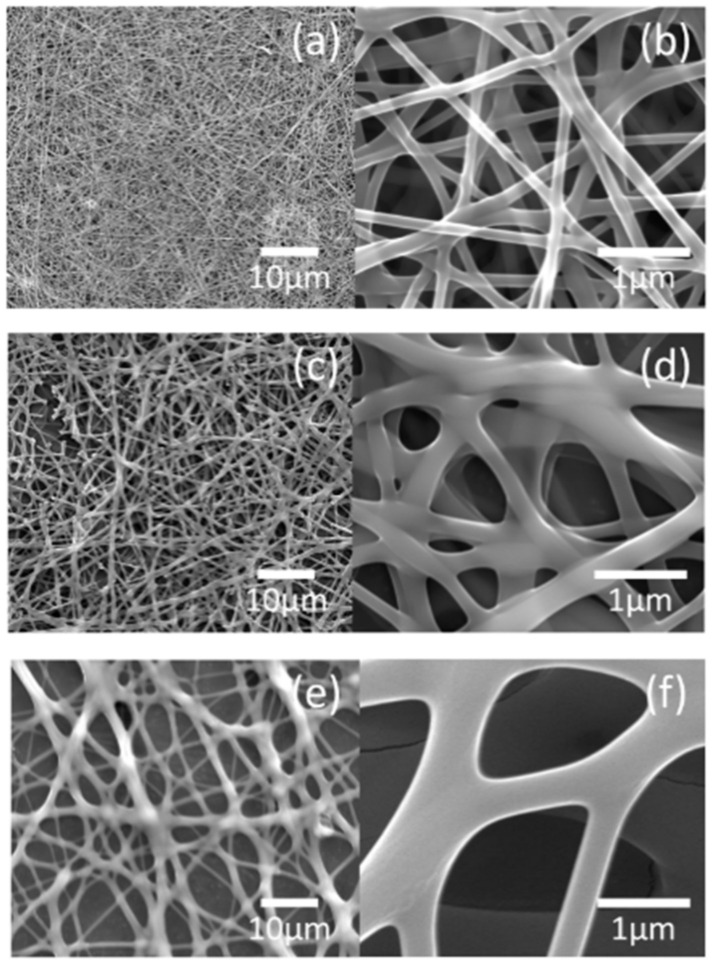
Field-emission scanning electron microscope (FE-SEM) images of as-spun nanofibers obtained from precursor solution 15-A (**a**) low magnification, (**b**) high magnification, as-spun nanofibers obtained from precursor solution 15-B (**c**) low magnification, (**d**) high magnification, and as-spun nanofibers obtained from precursor solution 15-C (**e**) low magnification, (**f**) high magnification.

Figure 3 shows the low- and high-magnification images of the as-spun nanofiber using ‘15-A’, ‘15-B’, and ‘15-C’ precursor solutions. Although the PVP content was constant and only the metal nitrate content was controlled, the average diameter increased in proportion to the viscosity characteristics of the solution. With the increase in the average diameter, the morphology of the samples gradually showed a diffused structure even after attachment to the collector. This was because, as the jet diameter on the electrospinning process increased, the distance and flight time for solvent evaporation became insufficient.

**Figure 4:**
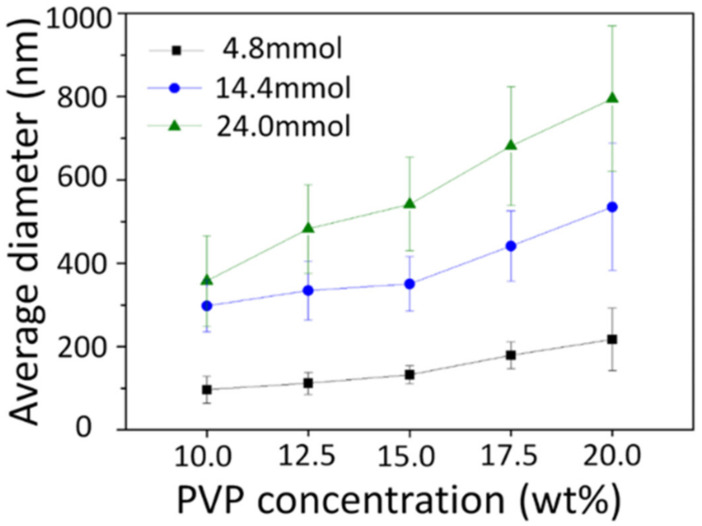
Average diameters of as-spun nanofibers obtained from each precursor solution.

Figure 4 shows the average diameter of as-spun nanofibers using each precursor solution. The diameter increased with increased PVP concentration and metal nitrate content. These factors, the PVP and metal nitrates, also increase the viscosity, and the average diameter was also affected by the viscosity of the precursor solution.

**Figure 5:**
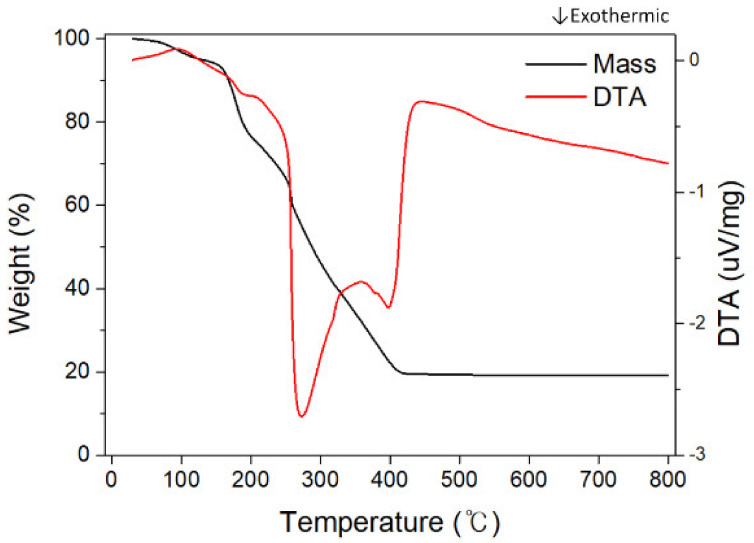
Thermogravimetric (TGA) curve of as-spun nanofibers obtained from precursor solution 15-B.

Figure 5 shows the TGA-DTA graph for the 15-B sample. The TGA-DTA data confirmed that there are four endothermic and exothermic reactions during the heat treatment process, and each TGA mass reduction section can be explained based on the DTA peak. The first mass reduction occurs gradually from 25 °C to 144 °C, which indicates the evaporation of adsorbed moisture and residual solvent. The DTA peak is located at 100 °C and is an endothermic reaction for the transition from liquid to a gas phase. Thereafter, there is a continuous exothermic reaction that is caused by PVP degradation, and then the rate of mass reduction decreases, and the slope of the DTA increases. The DTA peak is located at around 200 °C, and can be explained by the glass transition of the PVP. The known glass transition temperature of pure PVP is from 180 °C to 200 °C, and close to the secondary DTA peak [27]. The thermal reaction temperature is variable due to the molecular weight of PVP and the nitrate input. The glass transition is an endothermic reaction, and the continuous exothermic reaction is temporally suppressed due to this reaction. Thereafter, rapid mass loss resumes at about 257 °C, and the DTA peak of this reaction is at 276 °C. This can be thought of as the ignition of PVP. It is a lower temperature than the known ignition point of pure PVP, but it may be that the ignition point shifted to a lower temperature due to the high thermal conductivity of the contained metal ions [28]. Subsequently, mass reduction continues and there is a final exothermic peak around 400 °C, which is likely the crystallization of Ni_0.5_Zn_0.5_Fe_2_O_4_ spinel ferrite after the degradation of residual carbon black and volatile components. In this reaction, a very small decrease in the mass reduction rate was measured, but it was not significant and can be considered to be the oxygen absorption from the oxidation and crystallization. The results of the TGA-DTA measurement showed that all thermal reactions were finished below 420 °C, but considering the confounder and safety factors in the actual process, the calcination temperature was determined to be 650 °C.

The results of the TGA analysis of samples 15-A, 15-B, and 15-C are shown in Table 2. The obtained quantity of calcined nanofibers tended to increase according to inputs of the metal nitrates, and the total mass reduction decreased to 77.57 from 84.02 with metal nitrates. Since all as-spun nanofibers arrived at the final product through a heat treatment process, when the same amount of as-spun nanofiber was heat treated, the mass of the final product was increased from 15.98 wt% in 15-A to 22.43 wt% in 15-C. This means that the process yield for mass production was improved by 140% by increasing the amount of metal nitrates in 15-C.

### 3.3. Calcined Nanofibers

**Figure 6:**
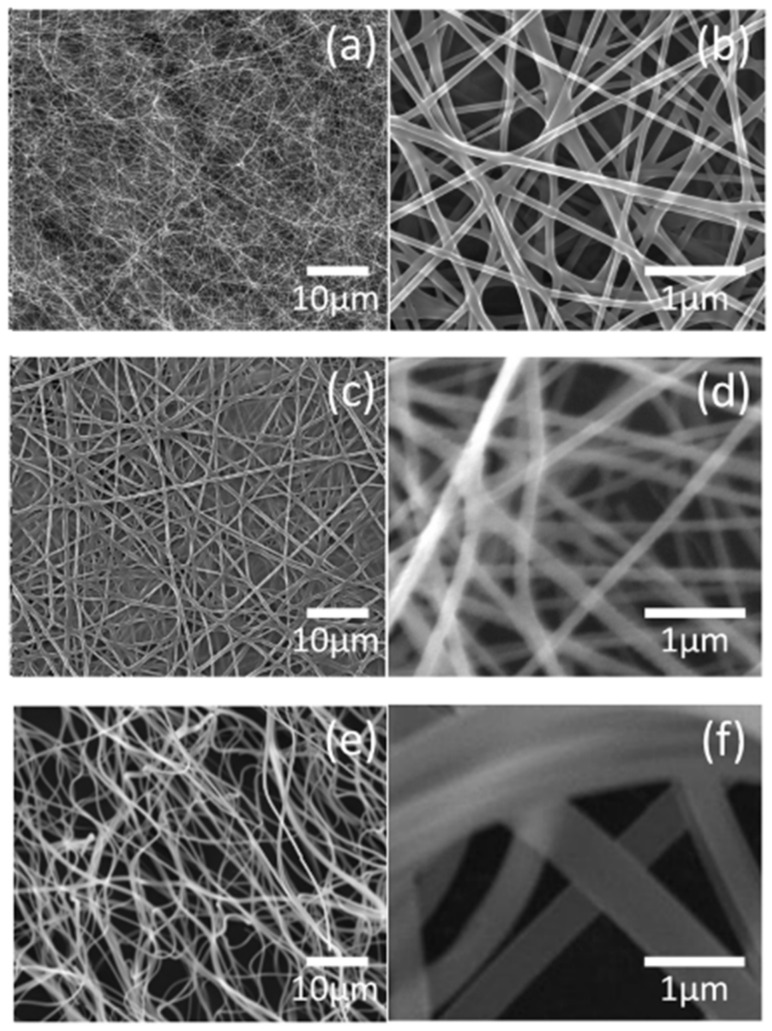
FE-SEM images of calcined Ni_0.5_Zn_0.5_Fe_2_O_4_ nanofibers obtained from precursor solution 15-A (**a**) low magnification, (**b**) high magnification, Calcined Ni_0.5_Zn_0.5_Fe_2_O_4_ nanofibers obtained from precursor solution 15-B (**c**) low magnification, (**d**) high magnification, and Calcined Ni_0.5_Zn_0.5_Fe_2_O_4_ nanofibers obtained from precursor solution 15-C (**e**) low magnification, (**f**) high magnification.

Figure 6 shows FE-SEM images of calcined nanofiber samples using precursor solutions 15-A, 15-B, and 15-C. It can be seen that the one-dimensional nanofibrous structure was well maintained even after the heat treatment, and the average diameter of the nanofibers was significantly reduced. In the 15-C sample with a high viscosity solution, which revealed the network structure, a clear fibrous structure was also expressed by the three-dimensional (3-D) contraction that centered on the fiber core. However, there were more broken and curved fibers compared with the samples using other precursor solutions with a lower viscosity.

**Figure 7:**
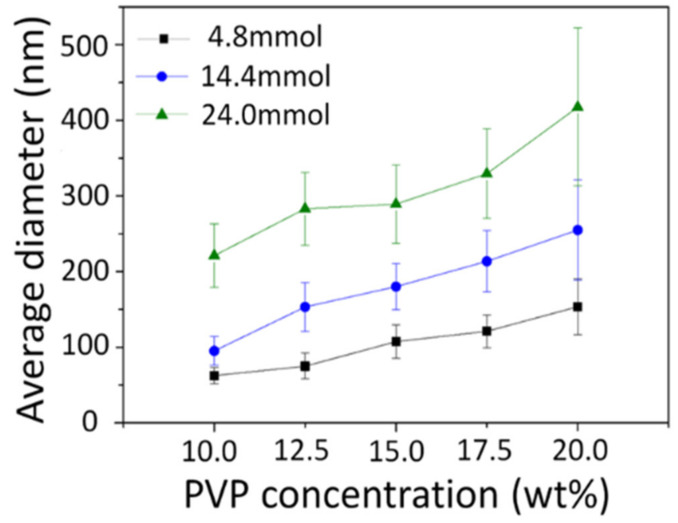
Average diameters of calcined Ni_0.5_Zn_0.5_Fe_2_O_4_ nanofibers obtained from each precursor solution.

Figure 7 shows the average diameter of calcined nanofibers analyzed by FE-SEM images, which confirmed that the average diameter was rapidly reduced after heat treatment, and that there was also an increasing tendency according to the amount of metal nitrates remaining after heat treatment.

**Figure 8:**
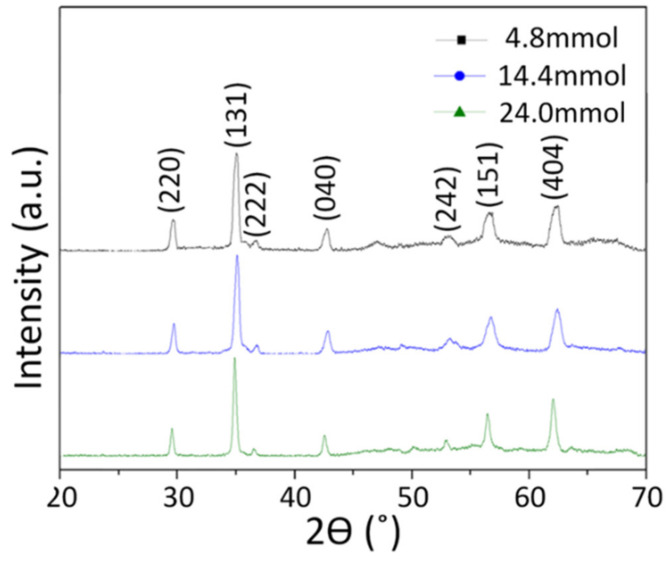
X-ray diffraction patterns of the calcined Ni_0.5_Zn_0.5_Fe_2_O_4_ nanofibers obtained from precursor solutions 15-A, 15-B, and 15-C.

The XRD analysis results of calcined nanofibers obtained using precursors 15-A, 15-B, and 15-C are shown in Figure 8. The significant peaks of the diffraction pattern were indexed with the (220), (131), (222), (040), (242), (151), and (404) planes in comparison with the JCPDS card 52-0278, and it was confirmed that the crystal phase of all the samples was Ni_0.5_Zn_0.5_Fe_2_O_4_ spinel ferrite.

The average crystallite sizes of the nanofibers were calculated using the Scherrer equation (D_p_ = 0.94 λ/β Cosθ). In this equation, the meaning of each variable is as follows: Dp = average crystallite size, β = full width at half maximum (FWHM) of the peak, θ = Bragg angle, and λ is the wavelength of the X-ray used for diffraction. The estimated crystallite size is shown in Table 3 and increases from 17.42 nm to 25.56 nm with nitrate content. As the nitrate content increases, the PVP content ratio decreases relatively, so this result is consistent with the report of Xiaolei Song et al. that high polymer content increases crystal defects and makes it difficult to grow primary grain [29], which means that an increase in nitrates improves not only the yield but also the crystallinity of the nanofibers. The FE-SEM image analysis and XRD results confirmed that there was no significant difference in the nanofibers obtained using metal nitrate content control.

## 4. Conclusions

In the DMF solution in which PVP was dissolved, adding metal nitrates caused a rapid increase in viscosity. This technique can be usefully applied to control the average diameter in the electrospinning process. The average diameter can be controlled using other variables including polymer content, but it is possible to increase the yield and economic efficiency of the process if the desired microstructure can be achieved using the metal nitrate content. Since the polymer used as a support in the electrospinning process is a component that is completely volatilized and consumed in the process, if the use of polymers is minimized and the metal nitrates increased, raw material costs are reduced and the yield of calcined nanofibers is increased. In 24.0 mmol of metal nitrate precursor solution, the process yield was improved to 140% after heat treatment. There was no significant difference in the microstructure of the nanofibers between the polymer-controlled nanofibers and the nitrate-controlled nanofibers to improve a process yield for mass production. This result confirms that NiZn ferrite nanofibers are available for mass production. NiZn ferrite nanofibers will be very useful for various applications, such as microwave absorbers, inductors, transformers, and radio frequency (RF) components.

## Figures and Tables

**Table 1:** The naming by composition and content of precursor solutions.

Name	Ni(NO_3_)_2_·6H_2_O (g)	Zn(NO_3_)_2_·6H_2_O (g)	Fe(NO_3_)_2_·9H_2_O (g)	PVP (g)	DMF (g)
10-A	0.23	0.24	1.29	10.0	90.0
10-B	0.70	0.71	3.88		
10-C	1.16	1.19	6.46		
12.5-A	0.23	0.24	1.29	12.5	87.5
12.5-B	0.70	0.71	3.88		
12.5-C	1.16	1.19	6.46		
15-A	0.23	0.24	1.29	15.0	85.0
15-B	0.70	0.71	3.88		
15-C	1.16	1.19	6.46		
17.5-A	0.23	0.24	1.29	17.5	82.5
17.5-B	0.70	0.71	3.88		
17.5-C	1.16	1.19	6.46		
20-A	0.23	0.24	1.29	20.0	80.0
20-B	0.70	0.71	3.88		
20-C	1.16	1.19	6.46		

**Table 2:** The thermal reaction classification of the TGA peaks.

Precursor Solution	Temperature Range (°C)	Weight Loss (%)	Total Mass Reduction (%)	Thermal Reaction
15-A	0–150	6.19	84.02	Endothermic
	150–240	19.58		Exo/endothermic
	240–270	19.74		Exothermic
	270–420	38.51		Exothermic
15-B	0–150	6.12	80.85	Endothermic
	150–240	24.99		Exo/endothermic
	240–270	13.56		Exothermic
	270–420	36.18		Exothermic
15-C	0–150	5.47	77.57	Endothermic
	150–240	24.08		Exo/endothermic
	240–270	15.19		Exothermic
	270–420	32.83		Exothermic

**Table 3:** The estimated average crystallite size of calcined Ni_0.5_Zn_0.5_Fe_2_O_4_ nanofibers.

Precursor Solution	Plane	2θ (°)	FWHM (°)	Average Crystallite Size (Diameter, nm)
15-A	(202)	29.69	0.37	17.42
	(131)	35.11	0.42	
	(404)	62.46	0.81	
15-B	(202)	29.97	0.36	19.19
	(131)	35.36	0.41	
	(404)	62.58	0.78	
15-C	(202)	29.64	0.25	25.56
	(131)	34.84	0.32	
	(404)	62.16	0.64	
